# Characterizing Patient-Reported Fatigue Using Electronic Diaries in Neurodegenerative and Immune-Mediated Inflammatory Diseases: Observational Study

**DOI:** 10.2196/65879

**Published:** 2025-05-05

**Authors:** Adrien Bennetot, Rana Zia Ur Rehman, Robbin Romijnders, Zhi Li, Victoria Macrae, Kristen Davies, Wan-Fai Ng, Walter Maetzler, Jennifer Kudelka, Hanna Hildesheim, Kirsten Emmert, Emma Paulides, C Janneke van der Woude, Ralf Reilmann, Svenja Aufenberg, Meenakshi Chatterjee, Nikolay V Manyakov, Clémence Pinaud, Stefan Avey

**Affiliations:** 1 Let it Care, 11 avenue Marquise du Deffand, Antony, 92160, France, 33 08 92 97 06 43; 2 Johnson & Johnson, Buckinghamshire, United Kingdom; 3 Department of Neurology, University Hospital Schleswig-Holstein Campus Kiel, Kiel University, Kiel, Germany; 4 Johnson & Johnson, Spring House, PA, United States; 5 NIHR Newcastle Biomedical Research Centre, Newcastle Upon Tyne Hospitals NHS Foundation Trust, Newcastle Upon Tyne, United Kingdom; 6 NIHR Newcastle Clinical Research Facility, Newcastle Upon Tyne Hospitals NHS Foundation Trust, Newcastle Upon Tyne, United Kingdom; 7 Translational and Clinical Research Institute, Faculty of Medical Sciences, Newcastle University, Newcastle Upon Tyne, United Kingdom; 8 HRB Clinical Research Facility, University College Cork, Cork, Ireland; 9 Department of Gastroenterology and Hepatology, Erasmus MC, Rotterdam, The Netherlands; 10 George-Huntington-Institute, University of Münster, Münster, Germany; 11 Department of Neurodegenerative Diseases, Hertie Institute for Clinical Brain Research , University of Tübingen, Tübingen, Germany; 12 Department of Clinical Radiology, University of Münster, Münster, Germany; 13 Johnson & Johnson, Cambridge, MA, United States; 14 Johnson & Johnson, Beerse, Belgium

**Keywords:** chronic disease, fatigue, neurodegenerative diseases, immune-mediated inflammatory diseases, diary, patient-reported outcomes, electronic diaries, digital technologies, digital health, eHealth, mobile phone

## Abstract

**Background:**

Fatigue is a prevalent and debilitating symptom in many chronic conditions, including immune-mediated inflammatory diseases (IMIDs) and neurodegenerative diseases (NDDs). Fatigue often fluctuates significantly within and between days, yet traditional patient-reported outcomes (PROs) typically rely on recall periods of a week or more, potentially missing these short-term variations. The development of digital tools, such as electronic diaries (eDiaries), offers a unique opportunity to collect granular, real-time data. However, the feasibility, adherence, and comparability of eDiary-based assessments to established PROs require further investigation.

**Objective:**

This study aimed to evaluate the feasibility and acceptability of using a high-frequency eDiary to capture intraday variability in fatigue and to compare eDiary data with scores obtained from the Functional Assessment of Chronic Illness Therapy-Fatigue (FACIT-F), a validated weekly recall PRO.

**Methods:**

Data were collected from 159 participants enrolled in the IDEA-FAST (Identifying Digital Endpoints to Assess Fatigue, Sleep and Activities in Daily Living in Neurodegenerative Disorders and Immune-Mediated Inflammatory Diseases) feasibility study; a 4-week prospective observational study conducted at 4 European centers. Participants included individuals with NDDs (n=39), IMIDs (n=78), and healthy volunteers (n=42). Participants used an eDiary to report their physical and mental fatigue levels up to 4 times daily on a 7-point Likert scale (0=low and 6=high). Adherence was calculated as the proportion of completed eDiary entries relative to the total expected entries. Correlations between averaged eDiary scores and weekly FACIT-F scores were analyzed.

**Results:**

Adherence to the eDiary protocol was 5505/8880 (61.99%) overall, varying by cohort, with the highest adherence (1117/1200, 93.07%) observed in the primary Sjögren syndrome cohort and the lowest adherence in the Parkinson disease (410/960, 42.7%) and Huntington disease (320/720, 44.4%) cohorts. The average adherence was 430/1680 (43.45%) in the NDD cohorts and 3367/4560 (73.84%) in the IMID cohorts. Fatigue levels showed clear diurnal variation, with significantly higher fatigue reported in the evening compared to the morning (*P*<.001). A moderate correlation (Spearman=0.46, *P*<.001) was observed between eDiary fatigue scores and FACIT-F scores, with stronger cohort-specific associations for certain FACIT-F items. These results indicate that eDiaries provide complementary insights to weekly PROs by capturing intraday fluctuations in fatigue.

**Conclusions:**

This study demonstrates the feasibility, acceptability, and validity of using high-frequency eDiaries to assess fatigue in chronic conditions. By effectively detecting intra- and interday fatigue variations, eDiaries complement traditional PROs such as FACIT-F, offering a more nuanced understanding of fatigue patterns. Future research should explore optimized eDiary protocols to balance participant burden with data granularity.

## Introduction

Fatigue poses a significant personal and financial burden to individuals living with immune-mediated inflammatory diseases (IMID) [1,2] and neurodegenerative diseases (NDD) [3] as it profoundly affects the individuals’ overall quality of life and daily activities [4]. The importance of fatigue is underscored by research priorities that emphasize the urgent need to unravel the intricate factors contributing to fatigue and to develop effective solutions [2].

Fatigue is a multifaceted phenomenon that extends beyond physical exhaustion, impacting cognitive, emotional, and motivational aspects [2,4]. Its complexity makes it challenging to assess and comprehend fully, particularly due to its subjective nature and variations among individuals, often not addressed during routine medical visits. Considering this, continuous monitoring of fatigue could provide useful information to facilitate fatigue assessment. By continuously observing and studying this complex phenomenon, we may also gain valuable insights into its variability and underlying mechanisms of fatigue.

Conventionally, participant-reported outcomes (PROs) have been the primary method of measuring fatigue in clinical trials and research studies. These PROs rely on self-reports to gauge the frequency, duration, and severity of fatigue [5,6]. However, PROs have several limitations, including recall bias and insensitivity to changes over time [7,8].

To address this need, an Innovative Medicines Initiative funded project was created called IDEA-FAST (Identifying Digital Endpoints to Assess Fatigue, Sleep and Activities in Daily Living in Neurodegenerative Disorders and Immune-Mediated Inflammatory Diseases) [9]: identifying digital end points to assess fatigue, sleep, and activities of daily living using a combination of digital health technologies to comprehensively assess fatigue in the real world. These digital measures hold the potential to provide objective assessments of fatigue that may complement self-reported fatigue to provide a more holistic view of fatigue. However, the identification and evaluation of objective digital measures of fatigue necessitates comparisons with perceived fatigue at relatively frequent intervals, preferably multiple times a day [10,11].

As such, this study focuses on evaluating the properties of a 4-times-a-day digital questionnaire. This study’s objective is to lay the foundation for the development and evaluation of digital measures of fatigue by examining their properties and comparing them with participant-reported fatigue.

In summary, this study aims to:

1. Determine whether there is any effect of time of the day (eg, morning, afternoon, and evening) on self-reported fatigue, which would argue in favor of fatigue assessments more often than once a day.
2. Determine if 4-times-a-day self-reported fatigue captures the overall trend of fatigue when compared to a validated questionnaire, such as the Functional Assessment of Chronic Illness Therapy-Fatigue (FACIT-F) [12-15] which has a 1-week recall period.
3. Determine the burden of participants to respond to an electronic diary (eDiary) for the assessment of fatigue 4 times a day for 4 weeks.

## Methods

### Ethical Considerations

The studies involving human participants were reviewed and approved by the Ethical Committee of the Medical Faculty, Kiel University, Kiel, Germany; Health Research Authority and Health and Care Research Wales, United Kingdom; Ethik-Kommission der Ärztekammer Westfalen-Lippe und der Westfälischen Wilhelms-Universität, Münster, Germany; and the Medical Ethics Review Committee, Erasmus MC, Netherlands. The patients or participants provided their written informed consent to participate in this study. Ethical approval was first granted by the Ethical Committee of the Medical Faculty of Kiel University (D491/20) in June 2020 and then by the Research Ethics Committees of all other study sites: Newcastle upon Tyne Hospitals National Health Service (NHS) Foundation Trust/Newcastle University in August 2020 (20/PR/0185), Erasmus University Medical Centre in Rotterdam in November 2020, and George-Huntington-Institute in Münster in September 2020 (2020-654-b-S). This study was registered with the German Clinical Trial Registry (DRKS00021693) and was conducted according to the principles of the Declaration of Helsinki (version of 2013). Data were pseudonymized in the clinical trial database before analysis.

Participants were recruited through multiple channels across the 4 study centers. Recruitment lists of individuals who had previously consented to be contacted for research purposes were used. Additionally, patients currently receiving inpatient or outpatient treatment at the respective clinics were informed about this study and invited to participate. Healthy control participants were recruited by approaching relatives of individuals with study-relevant diagnoses.

All participants received compensation for their travel expenses, which included coverage of costs incurred using public transport. For participants using their own car, reimbursement was provided based on the standard local cost per kilometer of travel. Importantly, no financial compensation was offered for participation in this study itself.

### Study Demographics

The feasibility study (FS) of the IDEA-FAST project aims to identify candidate digital measures of fatigue and sleep disturbances in 6 disease populations which include Parkinson disease (PD), Huntington disease (HD), inflammatory bowel disease (IBD), primary Sjögren syndrome (PSS), rheumatoid arthritis (RA), and systemic lupus erythematosus (SLE) as well as healthy volunteers.

Participants from the 7 cohorts (healthy, PD, HD, IBD, SLE, RA, and PSS) were recruited in 4 European centers: Universitätsklinikum Schleswig-Holstein Kiel, Newcastle University, Erasmus University Medical Centre Rotterdam, and George-Huntington-Institute Münster. A total of 159 volunteers were recruited for this study. Among them, 111 participants filled at least 2 eDiary entries and 128 participants completed at least 1 “home visit,” during which a staff nurse would visit them at home or call them on the telephone to make them fill in the weekly PROs. Participants at the intersection of these 2 sets are used for the analysis (103 participants) unless otherwise indicated.

Demographic and clinical aspects of the participants are summarized in Table 1.

**Table 1.** Demographics and clinical aspects of the recruited participants.

Variable and category		Participants		
		with clinical data (n=159)	with eDiary^a^ data (n=111)	with weekly PROs^b^ (n=128)
Age (years), mean (SD)		53 (16)	53 (16)	54 (15)
BMI, mean (SD)		26 (5)	26 (5)	26 (5)
Time since diagnosis (years), mean (SD)		11 (8)	11 (7)	11 (8)
Site, n (%)				
	Erasmus University Medical Centre Rotterdam	28 (18)	17 (15)	16 (13)
	George-Huntington-Institute	18 (12)	12 (11)	18 (14)
	Universitätsklinikum Schleswig-Holstein Kiel	56 (35)	34 (31)	41 (32)
	Newcastle University	57 (35)	48 (43)	53 (41)
Cohort, n (%)				
	HD^c^	14 (9)	7 (6)	12 (9)
	Healthy	42 (27)	30 (27)	38 (29)
	IBD^d^	18 (11)	13 (11)	9 (7)
	PD^e^	25 (16)	16 (15)	18 (15)
	PSS^f^	18 (11)	16 (15)	18 (15)
	RA^g^	24 (15)	16 (15)	17 (13)
	SLE^h^	18 (11)	13 (11)	16 (12)
Gender, n (%)				
	Female	102 (64)	74 (66)	82 (64)
	Male	57 (36)	37 (34)	46 (36)
Comorbidities, n (%)				
	At least 1 comorbidity	40 (25)	26 (23)	28 (21)
Physical activity (Godin^i^), n (%)				
	Active	90 (56)	70 (63)	79 (61)
	Insufficiently active	23 (14)	16 (14)	20 (15)
	Moderately active	24 (15)	19 (17)	22 (17)

a eDiary: electronic diary.

b PRO: participant-reported outcome.

c HD: Huntington disease.

d IBD: inflammatory bowel disease.

e PD: Parkinson disease.

f PSS: primary Sjögren syndrome.

g RA: rheumatoid arthritis.

h SLE: systemic lupus erythematosus.

i Godin: Godin-Shephard leisure-time physical activity questionnaire.

### Protocol and Data Collection

**Figure 1.**
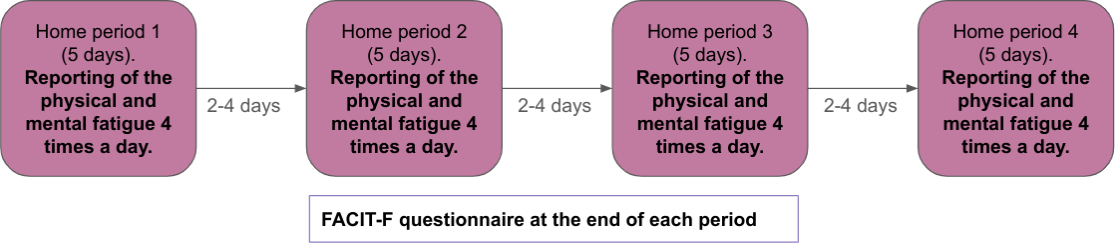
Data collection protocol for this study, composed of 4 home periods of 5 days spaced between 2 and 4 days. During each period participants reported their physical and mental fatigue 4 times a day. After each period, participants completed a FACIT-F questionnaire. FACIT-F: Functional Assessment of Chronic Illness Therapy-Fatigue.

Participants attended a scheduled study visit at the recruitment center at the beginning of this study, during which their basic demographics and medical data were collected. Each participant underwent a longitudinal evaluation over 4 weeks as described in Figure 1.

During this study, 2 types of data were collected:

- After each device use period, participants were asked to complete PROs, including the FACIT-F questionnaire, to assess their fatigue level during the past 7 days during a home visit or a phone call. The FACIT-F is a 13-item scale measuring fatigue, validated for several chronic illnesses including RA [14]. For each item, participants select a response from a 5-point Likert scale. Total scores range from 0 to 52 with lower scores indicating more fatigue [16].
- During the 4 weeks of this study, participants were asked to report their mental and physical levels of fatigue 4 times a day, by answering the question “At the moment I feel” with a 7 item Likert scale from “low” (0) to “high” (6). Participants must answer 2 times, once for physical fatigue and once for mental fatigue. Online questionnaires in the FS were facilitated by an app running on the participants’ smartphones. We refer to those questionnaires as the eDiary.

### Participant Adherence to the Protocol

Participant adherence to the 4-times-a-day eDiary was defined as the number of completed physical fatigue diary entries received during the first 28 days of the study divided by the total number of expected fatigue diary entries across all patients and days. The expected number of diary entries was calculated per patient as 80; four times a day for four 5-day home reporting periods. This method was chosen because the exact dates of the home period start and end were not recorded and varied by each participant. Adherence was calculated in the set of 111 participants with at least 1 eDiary entry. Adherence was first summarized at the participant level and then further aggregated by cohort.

### Effect of Time of the Day on Fatigue

The relationship between the level of fatigue and time of the day was investigated using linear mixed-effects models (LMMs), which can accommodate missing data [17], on the 4 times a day eDiary answers. Either physical or mental fatigue was set as the dependent variable, time of the day and cohort, and their interaction term, were modelled as fixed effects, and the subject was modelled as a random effect. As the eDiary responses were timestamped at the whole hour (eg, 9 am or 1 pm), the time of the day was modelled as a categorical variable (morning, afternoon, evening, and night). LMMs were calculated with JASP (Jeffreys’s Amazing Statistics Program), and an effect was considered significant if the corresponding *P* value of the likelihood ratio test was below .05.

### Daily eDiary Association With FACIT-F

Physical and mental fatigue from daily eDiary were compared with the FACIT-F overall score and its items. To examine the relationship between fatigue levels measured by the FACIT-F questionnaire and daily diary scores across the 4 home visits, a repeated-measures correlation analysis was conducted between FACIT-F and its items scores with a separately daily eDiary, where the eDiary score was averaged across each home period before FACIT-F assessment. This analysis accounts for the repeated nature of the data, which involved measuring fatigue at multiple time points for each participant.

Additionally, an association between each home visit and FACIT-F was also explored separately, where Spearman correlation coefficients were calculated to assess the strength and direction of the relationship between the FACIT-F questionnaire items and the average daily diary scores for physical and mental fatigue for each home visit separately. The significance level was set at *P*<.05.

Due to the exploratory nature of our analysis, no adjustments in multiple comparisons were performed.

## Results

### Participant Adherence

**Figure 2.**
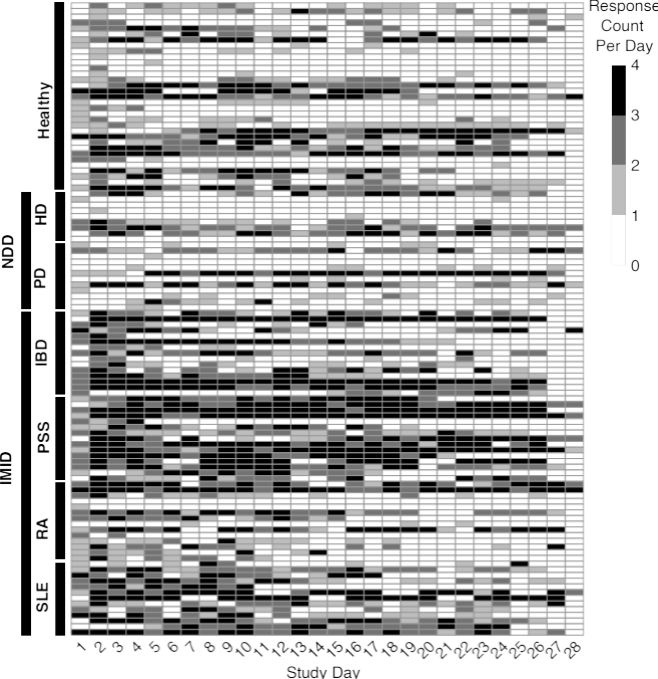
Heat map showing the number of completed eDiary physical fatigue questions per day (0‐4) with each study participant represented by 1 row. Note that there were 3 scheduled breaks of 2‐4 days between each of the four 5-day home periods during which no responses were expected. eDiary: electronic diary; HD: Huntington disease; PD: Parkinson disease; IBD: inflammatory bowel disease; IMID: immune-mediated inflammatory diseases; NDD: neurodegenerative diseases; PSS: primary Sjögren syndrome; RA: rheumatoid arthritis; SLE: systemic lupus erythematosus.

Overall, the adherence was acceptable given the high frequency of administration (4 times per day) with 5505/8880 (61.99%) of expected diary entries completed over all days. The percentage of daily responses completed by cohort and overall is shown in Figure 2. Across all participants, 88/111 (79.3%) of days had at least 1 response, 72/111 (64.9%) has at least 2 responses, 55/111 (49.5%) had at least 3 responses, and 32/111 (28.8%) had all 4 expected responses. The adherence varied dramatically between cohorts with the highest adherence (1117/1200, 93.08%) observed in the PSS cohort and the lowest adherence in the PD (410/960, 42.7%) and HD (320/720, 44.4%) cohorts. The average adherence was 730/1680 (43.45%) in the NDD cohorts and 3367/4560 (73.84%) in the IMID cohorts.

**Figure 3.**
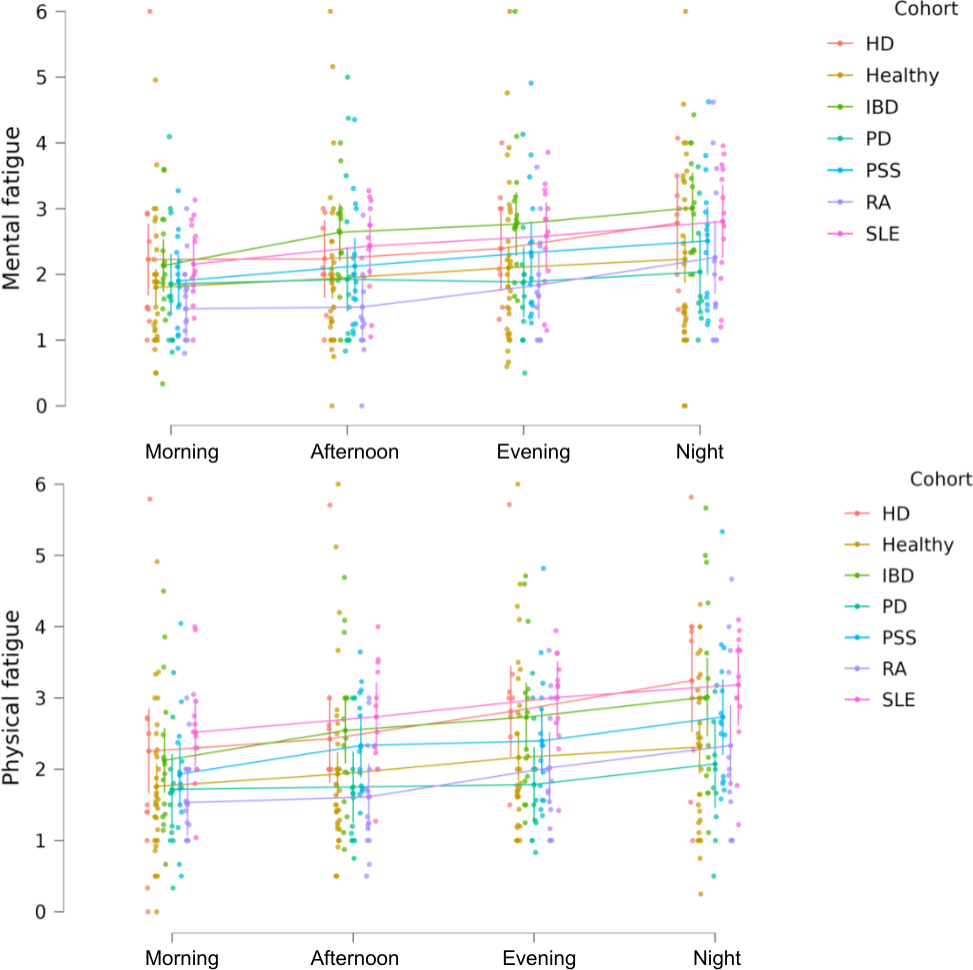
Scatterplot of subject-averaged physical and mental fatigue for different times of the day. Each dot represents the average self-reported fatigue for all responses for a single participant, during a respective period of a day. The cohort-level averages show increased levels of both physical (top) and mental fatigue (bottom) at later times of the day. The different cohorts are HD, healthy volunteers, IBD, PD, PSS, RA, and SLE. HD: Huntington disease; IBD: inflammatory bowel disease; PD: Parkinson disease; PSS: primary Sjögren syndrome; RA: rheumatoid arthritis; SLE: systemic lupus erythematosus.

For the following analyses we retained only those participants who responded to at least 1 FACIT-F questionnaire and at least 1 eDiary during 5 days before the FACIT-F. For the LMM, the likelihood ratio test showed that there were significant associations between time of the day and both physical fatigue and mental fatigue (Table 2). Visual inspection suggests that participants experienced more physical and mental fatigue at later times of the day (Figure 3).

**Table 2.** Results of LMMs^a^ used to assess the relationship between self-reported physical and mental fatigue levels and the time of day across 7 participant cohorts in the IDEA-FAST^b^ feasibility study.^c^

Effect		Chi-square (*df*)	*P* value
Physical fatigue			
	Cohort	17.3 (6)	.008
	Time of the day	54.2 (3)	<.001
	Cohort * time of the day	17.1 (18)	.51
Mental fatigue			
	Cohort	11.7 (6)	.07
	Time of the day	46.02 (3)	<.001
	Cohort * time of the day	20.4 (18)	.31

a LMM: linear mixed-effects model.

b IDEA-FAST [9]: Identifying Digital Endpoints to Assess Fatigue, Sleep and Activities in Daily Living in Neurodegenerative Disorders and Immune-Mediated Inflammatory Diseases.

c Fatigue levels were measured using a 7-point Likert scale (0=low, 6=high) with 4 daily eDiary (electronic diary) entries (morning, afternoon, evening, and night) over a 4-week observation period. The models included time of day and participant cohort as fixed effects and individual participants as random effects to account for repeated measures. The 7 cohorts included healthy volunteers and participants with Parkinson disease (PD), Huntington disease (HD), inflammatory bowel disease (IBD), primary Sjögren syndrome (PSS), rheumatoid arthritis (RA), and systemic lupus erythematosus (SLE). The table highlights significant diurnal variations in fatigue and differences between cohorts.

### Daily eDiary Association With FACIT-F

**Figure 4.**
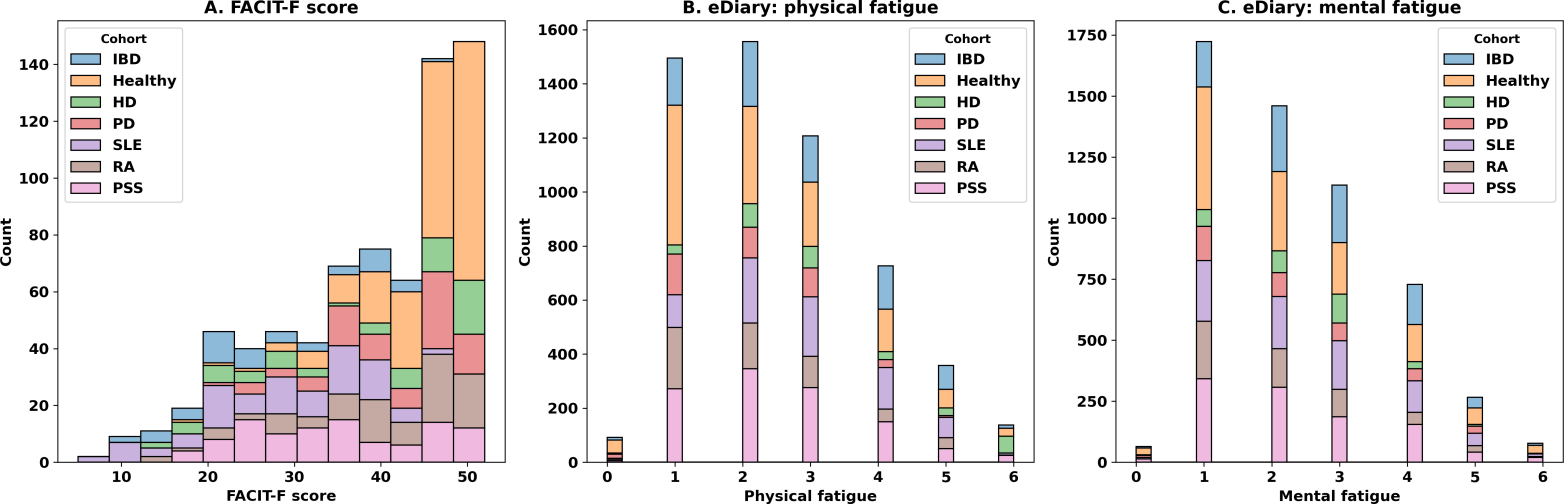
Distribution of responses. (A) Overall (total) FACIT-F score distribution, (B) physical fatigue from eDiary, and (C) mental fatigue from eDiary. Lower scores on the eDiary questions and higher scores on the FACIT-F questions reflect less fatigue. eDiary: electronic diary; FACIT-F: Functional Assessment of Chronic Illness Therapy-Fatigue; HD: Huntington disease; IBD: inflammatory bowel disease; PD: Parkinson disease; PSS: primary Sjögren syndrome; RA: rheumatoid arthritis; SLE: systemic lupus erythematosus.

The distributions of the physical and mental fatigue questions from daily eDiary and FACIT-F overall scores are shown in Figure 4A-C. Daily eDiary responses and FACIT-F scores are clustered around low fatigue levels.

**Figure 5.**
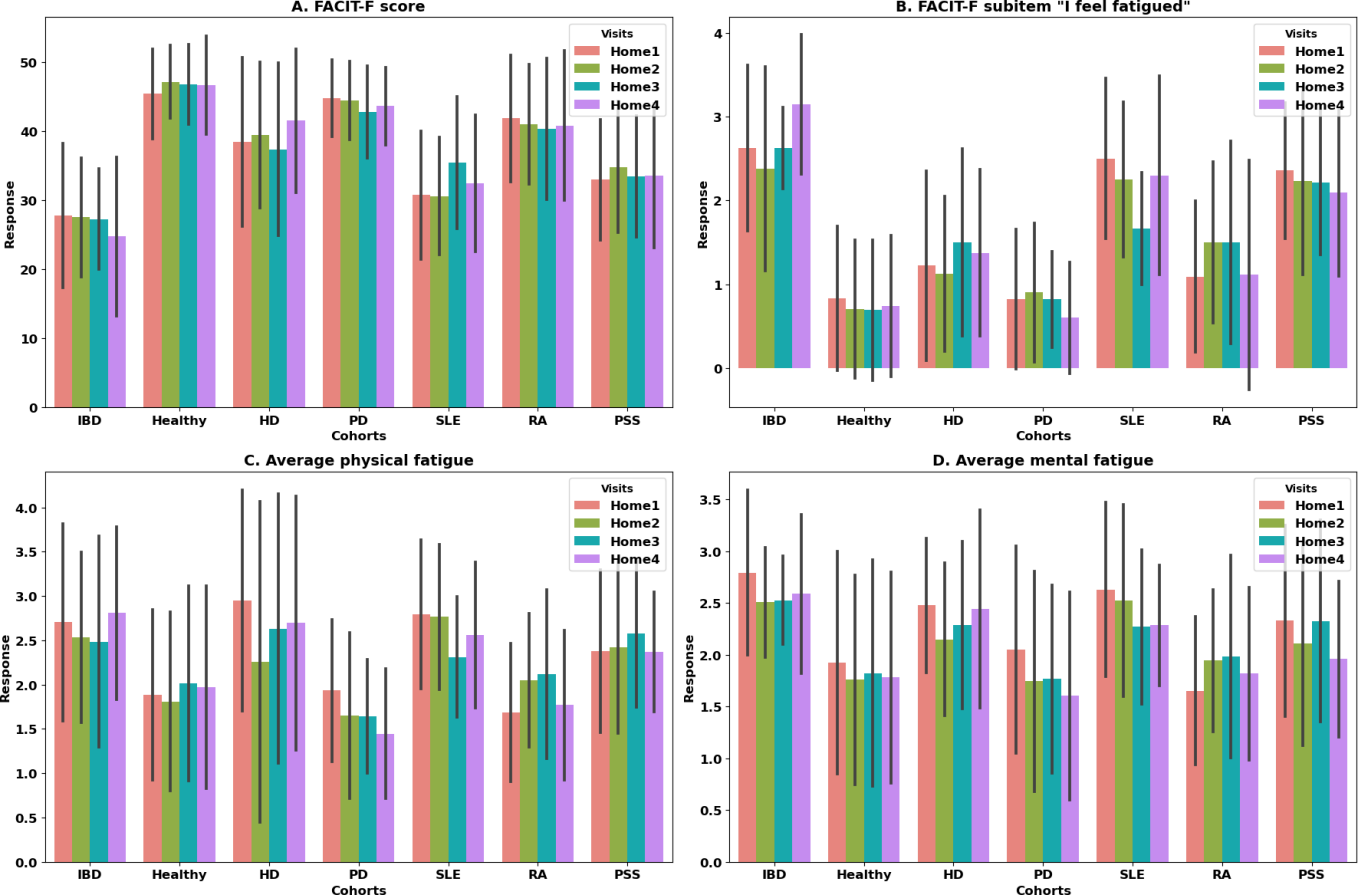
Responses from FACIT-F and daily diary across 4 home visits (bars indicate the average value while whiskers indicate the SD). (A) Bar plot of FACIT-F overall score (mean 39.57, SD 10.70), (B) bar plot of items of FACIT-F score: I feel fatigued (mean 1.42, SD 1.17), (C) daily dairy physical fatigue response average across 7 days before each home visit (mean 2.18, SD 1.06), and (D) mental fatigue response from daily diary average across 7 days before each home visit (mean 2.07, SD 0.96). The different cohorts are HD, healthy volunteers, IBD, PD, PSS, RA, and SLE. FACIT-F: Functional Assessment of Chronic Illness Therapy-Fatigue; HD: Huntington disease; IBD: inflammatory bowel disease; PD: Parkinson disease; PSS: primary Sjögren syndrome; RA: rheumatoid arthritis; SLE: systemic lupus erythematosus.

To observe the general trends in the eDiary and FACIT-F responses related to fatigue across various home visits within different cohorts, we plotted the average response of the FACIT-F score (Figure 5A) and the FACIT-F item “I feel fatigued” (Figure 5B) across each home visit as an example. This item “I feel fatigued” from the FACIT-F questionnaire (Figure 5B) is selected for the similarity of its wording to that of the eDiary. It exhibits a similar pattern within various cohorts (IBD, healthy, HD, PD, SLE, and RA) to the physical and mental fatigue eDiary 7-days average (Figure 5C, Figure 5D) before each home visit (corresponding to the end of home periods), where the higher scores in both eDiary and FACIT-F indicate higher fatigue levels. Among the cohorts studied, healthy volunteers demonstrated the lowest levels of fatigue, followed by the PD and HD cohorts.

**Figure 6.**
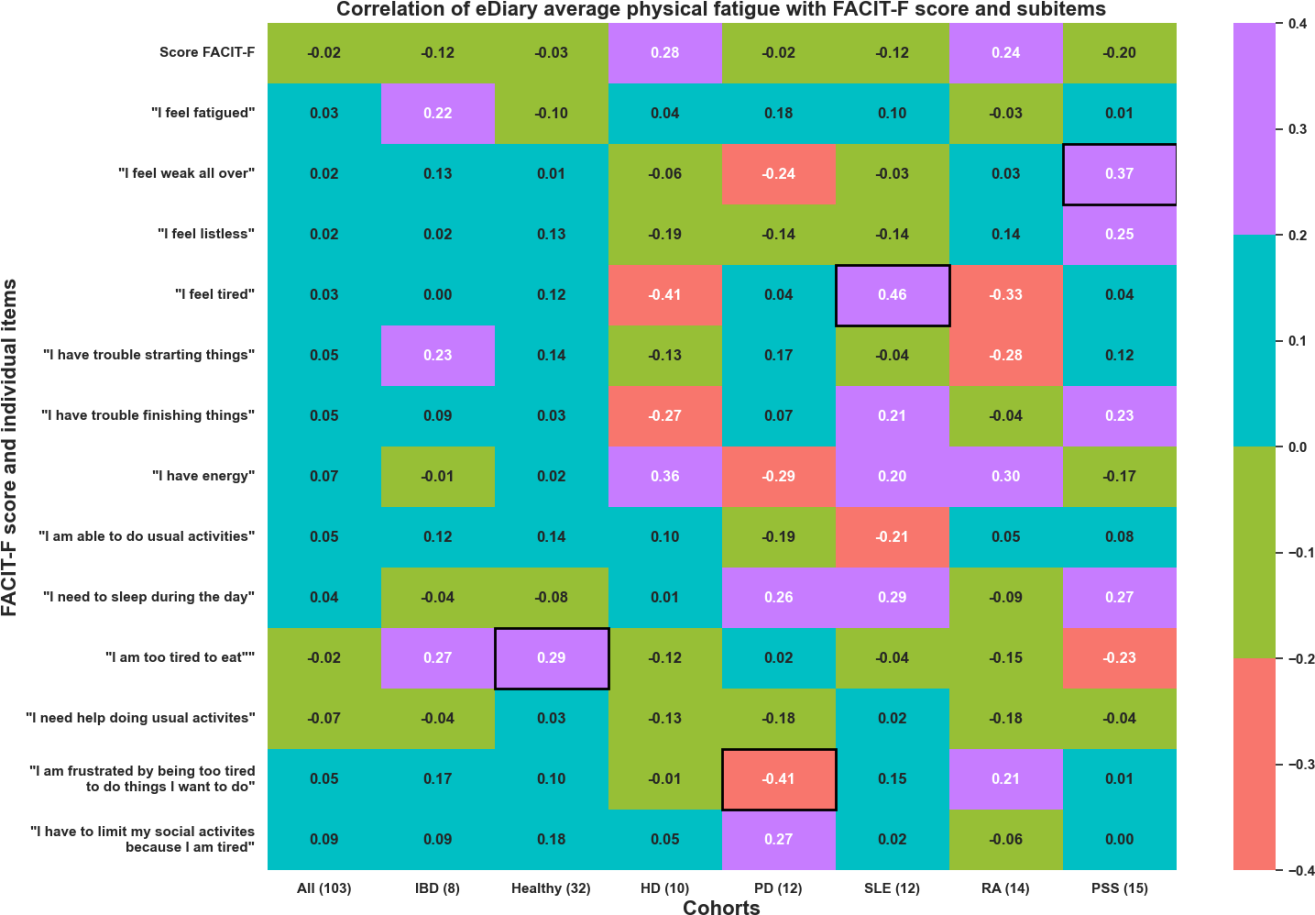
Repeated measure correlation between average daily physical fatigue measure with eDiary and FACIT-F overall score and its items. Values reaching statistical significance (*P*<.05) squared with a black box. Numbers in brackets, for example, “HD (10),” represent the number of different participants involved in the correlation calculation. The different cohorts are HD, healthy volunteers, IBD, PD, PSS, RA, and SLE. eDiary: electronic diary; FACIT-F: Functional Assessment of Chronic Illness Therapy-Fatigue; HD: Huntington disease; IBD: inflammatory bowel disease; PD: Parkinson disease; PSS: primary Sjögren syndrome; RA: rheumatoid arthritis; SLE: systemic lupus erythematosus.

Along with the total FACIT-F score, its item correlations with the eDiary were also investigated. Positive correlations are expected between FACIT-F items and eDiary but negative correlations are expected between the total FACIT-F score and the eDiary, as some items are inverted for the calculation of the total FACIT-F score [15]. Overall, a moderate repeated measure correlation was observed between several items of the FACIT-F questionnaire and the average daily eDiary scores for physical fatigue (Figure 6) and mental fatigue (not shown). However, only correlations between specific FACIT-F items and eDiary score reached statistical significance. For instance, in the PSS cohort, a positive correlation was found between the item “I feel weak all over” and both physical and mental fatigue. Other significant correlations are marked by the black boxes.

When combining all the cohorts, a negative insignificant correlation was observed between the overall FACIT-F score and both physical and mental fatigue averaged across 7 days before each home visit. Furthermore, certain items, including “feeling fatigued,” “feeling weak all over,” “feeling listless,” “feeling tired,” “experiencing trouble in starting and finishing tasks,” “having low energy,” and others, each exhibited a positive insignificant correlation with both the average physical and mental fatigue scores obtained from the daily eDiary.

**Figure 7.**
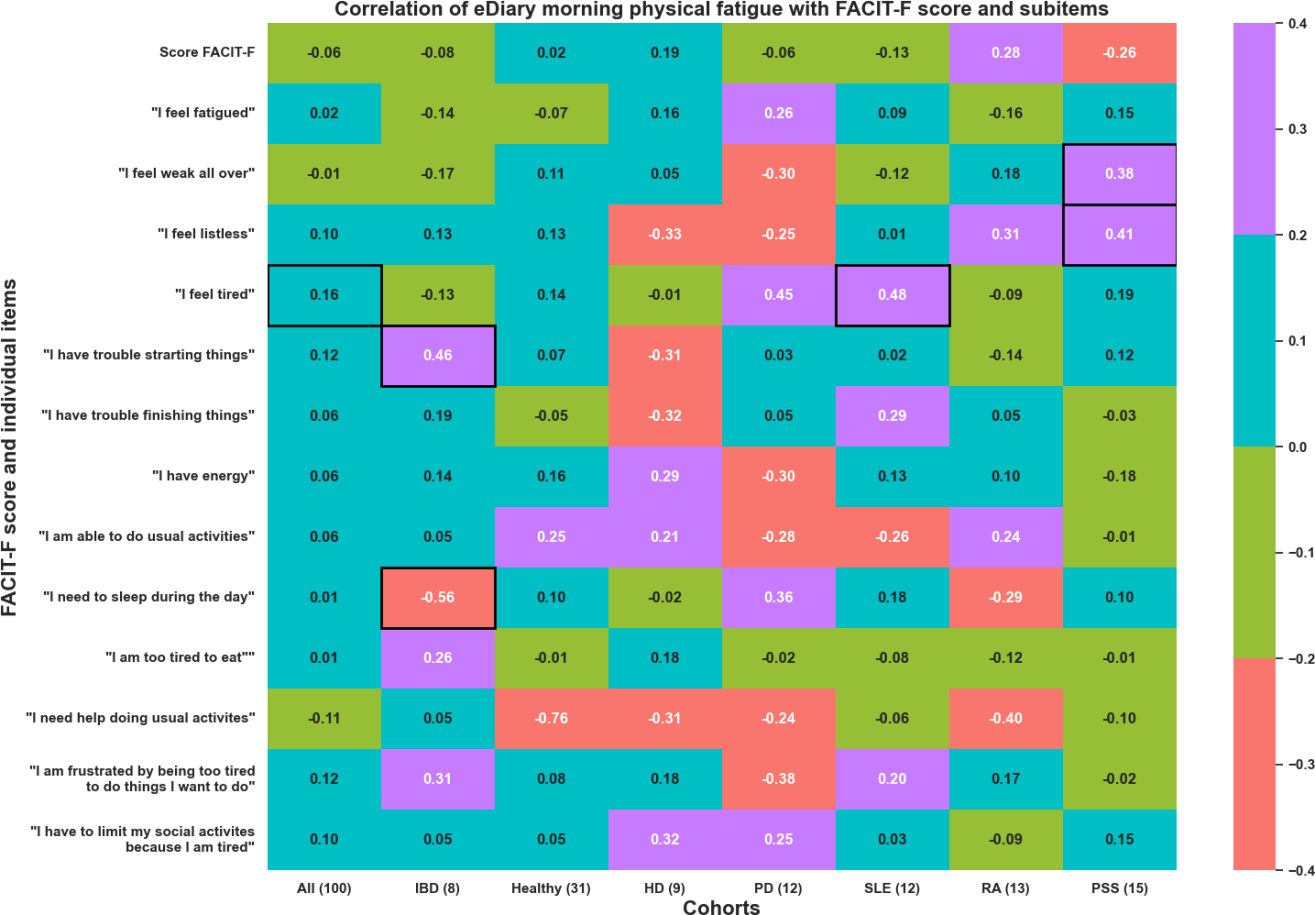
Repeated measures correlation between FACIT-F overall score and its items with 5-days averaged morning assessment of physical fatigue with the eDiary. The different cohorts are HD, healthy volunteers, IBD, PD, PSS, RA, and SLE. eDiary: electronic diary; FACIT-F: Functional Assessment of Chronic Illness Therapy-Fatigue; HD: Huntington disease; IBD: inflammatory bowel disease; PD: Parkinson disease; PSS: primary Sjögren syndrome; RA: rheumatoid arthritis; SLE: systemic lupus erythematosus.

Furthermore, the association between the eDiary at different time points (morning, early afternoon, late afternoon, and evening, which were averaged across the week before each home visit), and the FACIT-F score was explored. The heat maps in Figures7  8 illustrate the significant correlations observed in these weekly averaged measures in the morning and evening for physical fatigue (other periods of the day and mental fatigue in Multimedia Appendices 1-3). Importantly, the items having a significant correlation in the morning differed from those in the evening, both for physical and mental fatigue. These findings suggest that fatigue is a multifactorial phenomenon that varies throughout the day as well.

**Figure 8.**
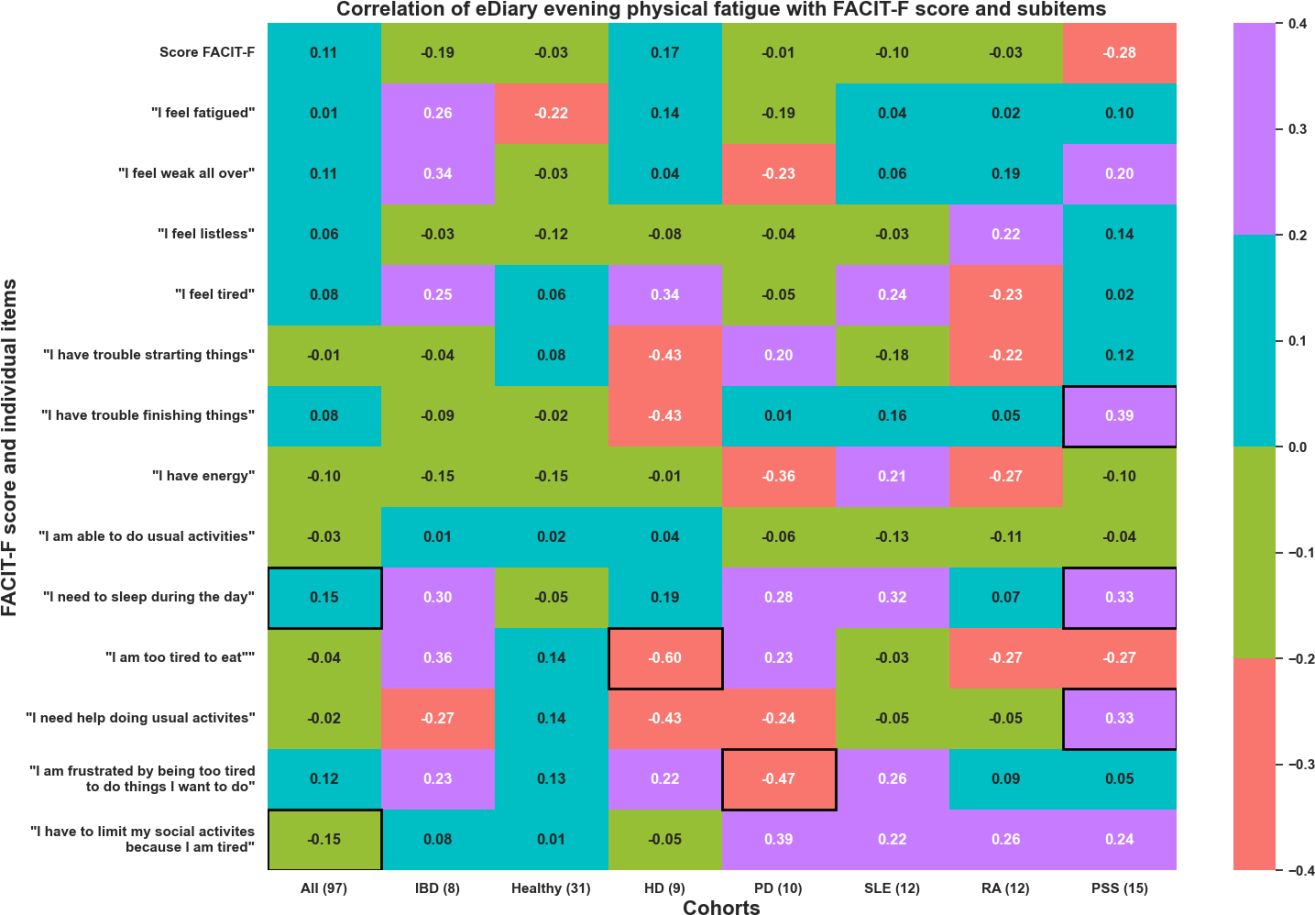
Repeated measures correlation between FACIT-F overall score and its items with the 5-days averaged evening assessment of physical fatigue with the eDiary. The different cohorts are HD, healthy volunteers, IBD, PD, PSS, RA, and SLE. eDiary: electronic diary; FACIT-F: Functional Assessment of Chronic Illness Therapy-Fatigue; HD: Huntington disease; IBD: inflammatory bowel disease; PD: Parkinson disease; PSS: primary Sjögren syndrome; RA: rheumatoid arthritis; SLE: systemic lupus erythematosus.

**Figure 9.**
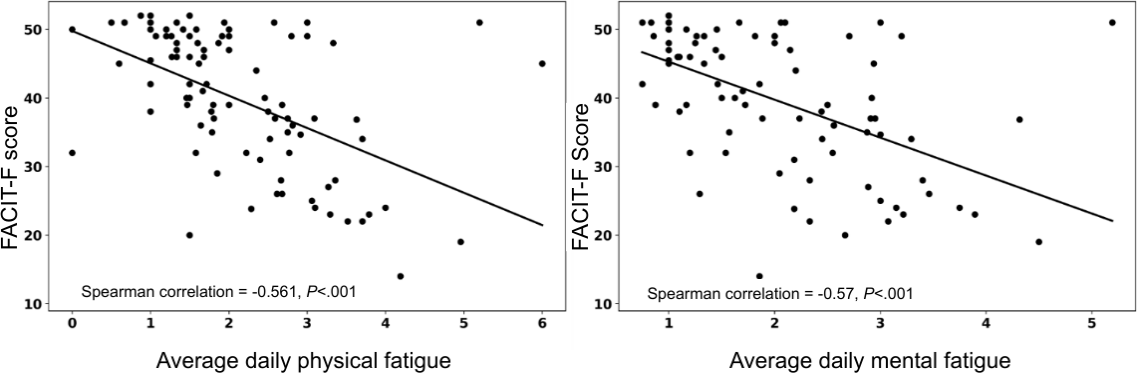
Scatter plot of the individual fatigue scores, with regression lines based on the Pearson correlation between the FACIT-F overall score and the average daily physical (left) and mental (right) fatigue for the home period 2. FACIT-F: Functional Assessment of Chronic Illness Therapy-Fatigue.

A significant negative Spearman correlation between the FACIT-F overall score and average eDiary score across each home period was observed separately for each visit. The correlation strength was in a similar range (from −0.4 to −0.6) across home visits for various cohorts. One example of the correlation is shown for home period 2 (Figure 9). Other visits are shown in Multimedia Appendices 1-3.

## Discussion

This study explored the feasibility of using a high-frequency eDiary for capturing intraday fluctuations in physical and mental fatigue among individuals with IMIDs, NDDs, and healthy volunteers. Our findings indicate that participants adhered to 5505/8880 (61.99%) of the expected diary entries over 4 weeks, with adherence varying across disease cohorts. Fatigue levels, both physical and mental, increased throughout the day, reflecting the impact of cumulative daily activity. This study demonstrated a moderate correlation between eDiary-reported fatigue scores and the FACIT-F, with stronger associations observed for specific cohorts and items, such as “I feel weak all over” in the PSS group. While overall significance across all groups was not achieved, the findings highlight the complementary role of eDiaries in capturing real-time fatigue patterns that align with weekly recall measures. The ability of eDiaries to detect intraday variability supports their use in fatigue modelling and personalized interventions, bridging subjective self-reports with broader clinical insights.

Overall, the average adherence (number of completed diary entries vs total number of expected diary entries) over the 4 weeks of the study was 5505/8880 (61.99%). Notably, the PSS cohort exhibited the highest percentage of completed eDiary responses, while the HD and PD groups had the lowest. A systematic review of the literature summarizing results from 12 studies in clinical contexts administering a mobile-collected ecological momentary assessment 4‐5 times/day for an average monitoring period of 12 days found that cohort compliance (defined as the percentage of participants in the cohort who were compliant) was approximately 75%‐85% [18]. This definition of adherence is different from our study, making a direct comparison challenging. It is important to consider that the participants’ involvement during our study extended beyond responding to the daily diary because participants of the FS were asked to use several wearable sensors, nonwearable sensors, and mobile phone apps. The complex protocol and participant burden of simultaneous device wearing might have contributed to the lower adherence observed in this study. Future studies should consider reducing the frequency to 3 times per day to retain the ability to capture intraday granularity while improving the probability of adherence. Future studies should also seek to understand how many times per day and over how many days fatigue should be measured to reliably assess fatigue symptoms before and after interventions. To investigate whether the time of the day influences self-reported levels of physical and mental fatigue, participants filled out an eDiary 4 times a day to report their level of fatigue on a scale from 0 (low fatigue) to 6 (high fatigue). Statistical analysis of the reported values by using an LMM suggests that physical and mental fatigue are positively correlated with the time of the day (*P* value<.001; Table 2). Physical fatigue and mental fatigue are considered 2 separate entities [19]. Physical fatigue is a form of tiredness caused by repeated muscle movements [20]. In contrast, mental fatigue is defined as a psychobiological state caused by prolonged periods of demanding cognitive activity [21,22]. The difference between physical and mental fatigue was clearly explained to the participants of our study and, as such, we can trust that they were able to discriminate them while filling in responses. The finding that at later times of the day higher levels of physical and mental fatigue are observed fits well with these definitions, as it can be expected that people have been both physically and mentally active during the day. The trend that at later times of the day more fatigue is experienced, is at least partly comparable with previous findings showing that tiredness fluctuates throughout the day in both a healthy and major depression disease cohort [23]. Similarly in healthy individuals, subjective tiredness was high in the morning, reached a trough at midday, and then rose steadily until the late evening time to resemble a V-shaped pattern [24,25]. The results presented in our study do not show a V-shape but a continuous increase in fatigue during the day. This difference could possibly be explained by that we are not modelling tiredness but fatigue, which are not the same entities [20,21,26], and the 7-point Likert scale has a limited range of possible values for fatigue. Overall, all these studies argue in favor of the presence of fatigue fluctuations throughout the day, supporting the need for continuous monitoring to capture intraday variability of fatigue.

One of the aims of this study was to assess whether self-reported fatigue reported 4 times a day accurately reflects the overall pattern of fatigue when compared to the validated FACIT-F questionnaire, which asks about fatigue over 1 week. The correlations between the daily eDiary entries and FACIT-F scores were examined in several participant cohorts, such as IBD, healthy, HD, SLE, RA, and PSS. Generally, persons who are in good health tend to have higher scores on the FACIT-F scale, which indicates that they experience less fatigue. On the other hand, participants with chronic diseases such as SLE and RA tend to have lower scores, indicating that they experience higher degrees of fatigue. The fatigue distribution exhibits a differentiation between the groups of individuals with good health and those with chronic illnesses. Concerning physical fatigue, individuals in good health report the lowest levels, while participants with SLE and RA experience higher levels. Participants with IBD and PSS exhibit moderate degrees of fatigue, but those with HD display varied amounts. Similarly, in the case of mental fatigue, those who are in good health report the lowest levels, while participants with SLE and RA report higher levels. Participants with HD, IBD, and PSS exhibit a wider range of levels, with many expressing considerable mental exhaustion. A moderate repeated measure correlation was found between specific items of the FACIT-F questionnaire and the average daily eDiary ratings for physical and mental fatigue. However, only few items achieved statistical significance. In the PSS cohort, a positive association was observed between the item “I feel weak all over” with both physical and mental exhaustion. When all groups were considered together, the overall association between FACIT-F scores and daily eDiary scores did not show statistical significance. However, specific items showed positive but insignificant relationships. This indicates that although eDiaries can accurately record patterns of fatigue, the degree of association may differ depending on the particular items and groups of participants.

These findings are consistent with previous research that shows the effectiveness of eDiaries in managing chronic illnesses [27] compared the FACIT-F with a simpler visual analogue scale ranging from 0 to 10 to evaluate fatigue. This study revealed a robust association between the 2 measures, indicating that frequent self-reports can effectively capture fatigue patterns observed in weekly evaluations. A separate investigation conducted by Cella et al [28] examined the extent to which the FACIT-F accurately measures self-reported fatigue ratings and found a strong level of consistency across several assessment techniques. Additionally, a study conducted by Rao et al [11] showed a good correlation between digital measures and daily self-reported exhaustion. This finding supports the reliability of frequent self-assessment in accurately capturing patterns of fatigue. There is a moderate correlation between daily eDiary entries and FACIT-F scores, suggesting eDiary could be used to monitor fatigue in near real time. This capacity improves patient care by allowing daily fatigue reports-based interventions. Four times a day fatigue data accurately represented fatigue trends, which may be compared to the FACIT-F questionnaire’s weekly recall period. The consistency of this pattern suggests that frequent self-reporting can provide reliable data for fatigue modelling using digital tools. Given the multifaceted nature of fatigue, a holistic approach to assessing the relevant physiological signals is essential, where the eDiary data captured during the day can act as a label.

Several limitations warrant consideration. First, this study’s complexity, involving eDiaries, wearable sensors, and multiple home visits, likely contributed to participant burden and reduced adherence. Simplified protocols could mitigate this issue. Second, the use of a 7-point Likert scale for fatigue assessment may have constrained variability, underestimating true intraday fluctuations. Third, this study’s duration of 4 times once a week may not reflect long-term patterns or the impact of seasonal changes. Additionally, participants frequently missed completing 1 or more fatigue questionnaires on a given day. This underscores the importance of optimizing assessment frequency to achieve a balance between maintaining data quality and ensuring participant adherence. Lastly, cohort-specific differences in adherence and correlations between eDiary and FACIT-F scores highlight the need for personalized approaches in future studies.

This study demonstrates the feasibility and utility of using high-frequency eDiaries for capturing intraday variations in physical and mental fatigue across diverse populations, including individuals with neurodegenerative and IMIDs. The observed adherence rates and correlations with the FACIT-F underscore the complementary value of eDiaries alongside established weekly recall-based measures. Importantly, eDiaries allow for the detection of nuanced diurnal patterns, providing a richer understanding of fatigue dynamics that traditional tools may overlook.

The integration of eDiaries into clinical research offers several broader implications. By enabling real-time monitoring, eDiaries can inform personalized interventions. These insights are particularly valuable for individuals with chronic conditions, where managing fatigue is a critical component of improving quality of life. From a research perspective, eDiaries can enhance the precision of clinical trials, allowing for better characterization of treatment effects on fatigue. Their ability to capture intraday fluctuations also opens avenues for exploring the interplay between fatigue and physiological markers captured by wearable sensors. This multimodal approach can deepen our understanding of fatigue’s multifactorial nature, bridging subjective experiences with objective measurements. However, the broader adoption of eDiaries necessitates attention to usability and adherence, particularly in populations with severe disease burdens or impairments. Simplified protocols, optimized frequency of assessments, and seamless integration with digital health ecosystems are critical for ensuring widespread adoption.

In conclusion, this study underscores the transformative potential of high-frequency digital tools in fatigue assessment. By complementing traditional measures with real-time insights, eDiaries have the potential to enhance fatigue management in clinical and research settings, paving the way for more personalized, data-driven approaches to chronic disease care.

## Supplementary material

10.2196/65879 Multimedia Appendix 1 Scatter plot of the Spearman correlation between the FACIT-F overall score and the average daily (left) physical and (right) mental fatigue for period 1. FACIT-F: Functional Assessment of Chronic Illness Therapy-Fatigue.

10.2196/65879 Multimedia Appendix 2 Scatter plot of the Spearman correlation between the FACIT-F overall score and the average daily (left) physical and (right) mental fatigue for period 3. FACIT-F: Functional Assessment of Chronic Illness Therapy-Fatigue.

10.2196/65879 Multimedia Appendix 3 Scatter plot of the Spearman correlation between the FACIT-F overall score and the average daily (left) physical and (right) mental fatigue for period 4. FACIT-F: Functional Assessment of Chronic Illness Therapy-Fatigue.

## Abbreviations

eDiary: electronic diary
FACIT-F: Functional Assessment of Chronic Illness Therapy-Fatigue
FS: feasibility study
HD: Huntington disease
IBD: inflammatory bowel disease
IDEA-FAST: Identifying Digital Endpoints to Assess Fatigue, Sleep and Activities in Daily Living in Neurodegenerative Disorders and Immune-Mediated Inflammatory Diseases
IMID: immune-mediated inflammatory disease
JASP: Jeffreys’s Amazing Statistics Program
LMM: linear mixed-effects model
NDD: neurodegenerative disease
PD: Parkinson disease
PRO: patient-reported outcome
PSS: primary Sjogren syndrome
RA: rheumatoid arthritis
SLE: systemic lupus erythematosus
