# Supplementary figures and images for: Characterizing Patient-Reported Fatigue Using Electronic Diaries in Neurodegenerative and Immune-Mediated Inflammatory Diseases: Observational Study
Source: JMIR Form Res. 2025 May 5;9:e65879. doi: 10.2196/65879 (PMC12068833; doi:10.2196/65879)

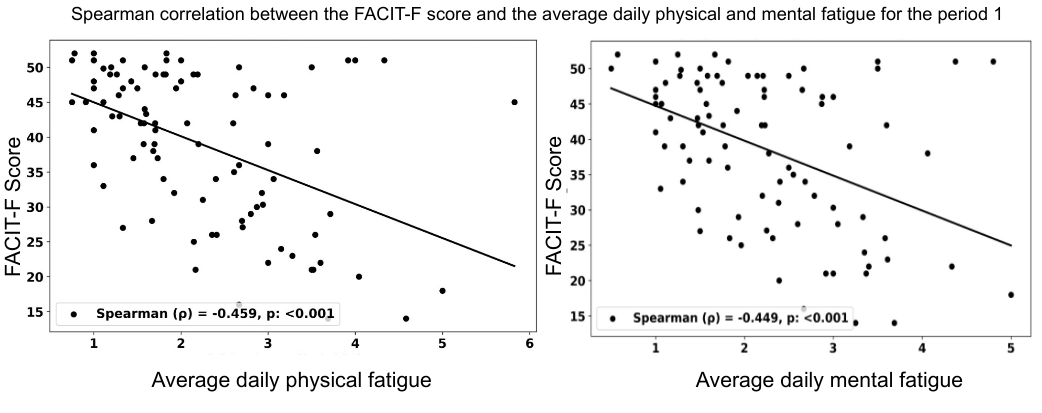

Supplement: Multimedia Appendix 1 [file formative-v9-e65879-s001.png]

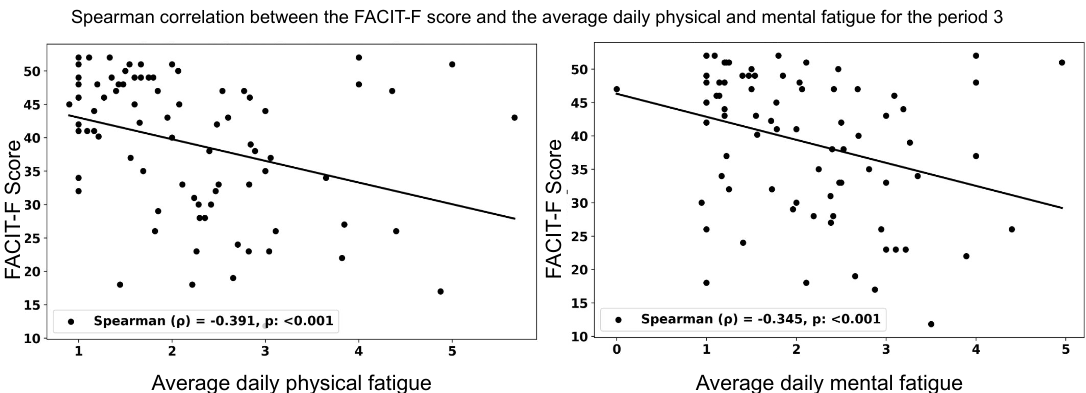

Supplement: Multimedia Appendix 2 [file formative-v9-e65879-s002.png]

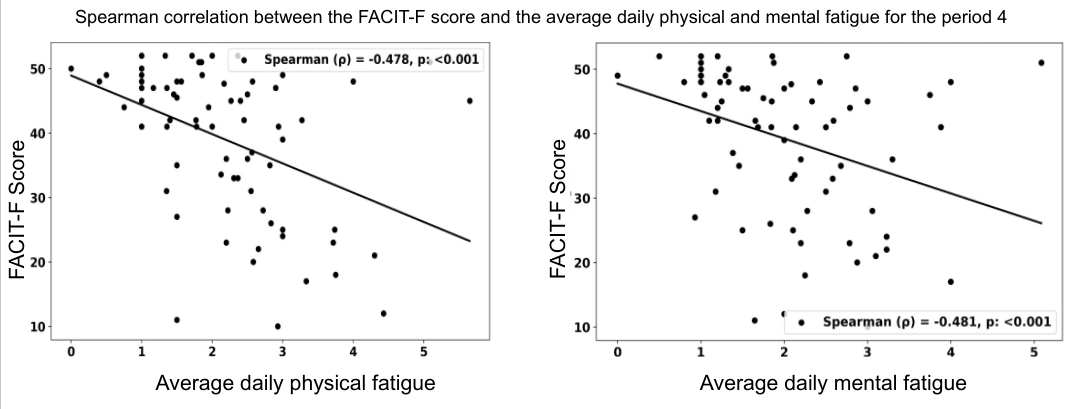

Supplement: Multimedia Appendix 3 [file formative-v9-e65879-s003.png]
